# Energetic, Structural and Dynamic Properties of Nucleobase-Urea Interactions that Aid in Urea Assisted RNA Unfolding

**DOI:** 10.1038/s41598-019-45010-8

**Published:** 2019-06-19

**Authors:** Tanashree Jaganade, Aditya Chattopadhyay, Nila M. Pazhayam, U. Deva Priyakumar

**Affiliations:** Center for Computational Natural Sciences and Bioinformatics, International Institute of Information Technology, Hyderabad, 500032 India

**Keywords:** Chemistry, Theoretical chemistry

## Abstract

Understanding the structure-function relationships of RNA has become increasingly important given the realization of its functional role in various cellular processes. Chemical denaturation of RNA by urea has been shown to be beneficial in investigating RNA stability and folding. Elucidation of the mechanism of unfolding of RNA by urea is important for understanding the folding pathways. In addition to studying denaturation of RNA in aqueous urea, it is important to understand the nature and strength of interactions of the building blocks of RNA. In this study, a systematic examination of the structural features and energetic factors involving interactions between nucleobases and urea is presented. Results from molecular dynamics (MD) simulations on each of the five DNA/RNA bases in water and eight different concentrations of aqueous urea, and free energy calculations using the thermodynamic integration method are presented. The interaction energies between all the nucleobases with the solvent environment and the transfer free energies become more favorable with respect to increase in the concentration of urea. Preferential interactions of urea versus water molecules with all model systems determined using Kirkwood-Buff integrals and two-domain models indicate preference of urea by nucleobases in comparison to water. The modes of interaction between urea and the nucleobases were analyzed in detail. In addition to the previously identified hydrogen bonding and stacking interactions between urea and nucleobases that stabilize the unfolded states of RNA in aqueous solution, NH-π interactions are proposed to be important. Dynamic properties of each of these three modes of interactions have been presented. The study provides fundamental insights into the nature of interaction of urea molecules with nucleobases and how it disrupts nucleic acids.

## Introduction

Understanding the underlying mechanism of folding and unfolding of biological macromolecules such as proteins and nucleic acids is essential, given their roles in cellular functions. Stabilities of these macromolecules are altered in the presence of various osmolytes, enzymes, and other denaturants^1^. Early studies demonstrated the importance of osmolytes in living organisms to cope with the osmotic pressure change^2^. Furthermore, a number of experimental studies have shown the role of osmolytes as denaturant or stabilizers depending on the nature of the osmolytes^2–5^. These experiments have played a crucial role in the understanding of protein stability and folding pathways. Urea is a polar molecule with a large dipole moment and has a strong effect on protein stability^6^. The underlying molecular mechanism behind urea-induced protein unfolding has been discussed extensively in the literature^4,7–10^. Two different mechanisms by which protein denaturation is achieved by aqueous urea have been proposed, namely, the direct and indirect mechanism. According to the direct mechanism, urea competes with the native intramolecular interactions within the protein structure^2,11,12^. The indirect mechanism suggests that urea alters the structure of water, which facilitates the weakening of the hydrophobic effect and destabilizes the native conformation^13^. Several experimental and computational studies have been performed to understand the structure, energetics, thermodynamic and mechanical aspects of urea assisted direct and indirect mechanism behind protein folding/unfolding^2,14–22^. Urea has also been shown to denature RNA, and these experiments provide insights into RNA stability and folding^15,23–26^. Unlike the mechanism of urea-induced protein denaturation, the effect of urea on nucleic acid unfolding is less understood. Several studies reported that urea induces significant changes in nucleic acid structures by interacting with the exposed surface of nucleobases, which subsequently results in the destabilization of nucleic acids^27–29^. Thermodynamic stabilities and folding kinetics studies have shown the importance of urea to examine nucleic acid stability^30–32^. Free energy analysis with m-value studies revealed urea forms stronger interactions with large surface areas of nucleobases that get exposed upon RNA denaturation compared to other regions of nucleic acids such as backbone, ribose, and phosphate^27^. Urea has been shown to preferentially solvate the nucleobases and helps in the denaturation process of nucleic acids while nucleic acid backbone has no major role in the denaturation process^28^. This implies urea-nucleobase interactions are crucial in understanding the mechanism by which the unfolded RNA is stabilized in the presence of urea than in the absence. Presence of stacking and hydrogen bonding interactions between urea and nucleobases contribute to the destabilization of nucleic acid structures^28,29,33–36^. These interactions have also been quantified using techniques like osmometry and hexanol-water distribution assays^27^. A recent quantum mechanical study reported that urea indeed forms strong stacking interactions with nucleobases dominated by dispersion^29^. The effect of urea on the overall structure of nucleic acids has been analyzed before^28,33,35^. However, how the interaction of each of the nucleobases with aqueous urea contributes towards stabilizing the RNA molecules in their unfolded states in the presence of aqueous urea is unclear. Earlier studies reported that the native state of the RNA initially gets disrupted by triggered penetration of water molecules, which results into destabilization of nucleobases^28,33^. The current study focuses on the mechanism by which the nucleobases of the unfolded RNA is stabilized by aqueous urea. Our main aim here is to understand what makes urea a most competent solvent to form stable interactions with nucleobases. This study is performed for an improved understanding of the effect of aqueous urea at individual base level (purines and pyrimidines) which helps to rationalize the contribution of these nucleobases towards stabilizing the unfolded state in the presence of urea. We have performed all-atom molecular dynamics simulations to assess the role of nucleobase-urea interactions and thermodynamic integration studies to assess the change in transfer free energies of the nucleobases from pure water to aqueous urea. This study gives molecular level explanations of effects of urea on nucleobases. Analyses from our simulations have allowed us to answer several questions: How urea forms stable interactions with nucleobases? How nucleobases preferentially interacts with urea than pure water? What are the solvation free energy changes involved in the interactions and which are the prominent interactions favored by urea while interacting with these nucleobases? This study explains the strength and nature of urea-nucleobase interactions and gives detailed insights to discern the mechanism of urea-induced nucleic acid disruption.

## Results and Discussion

### Presence of urea leads to favorable interaction with nucleobases primarily via dispersion

**Figure 1:**
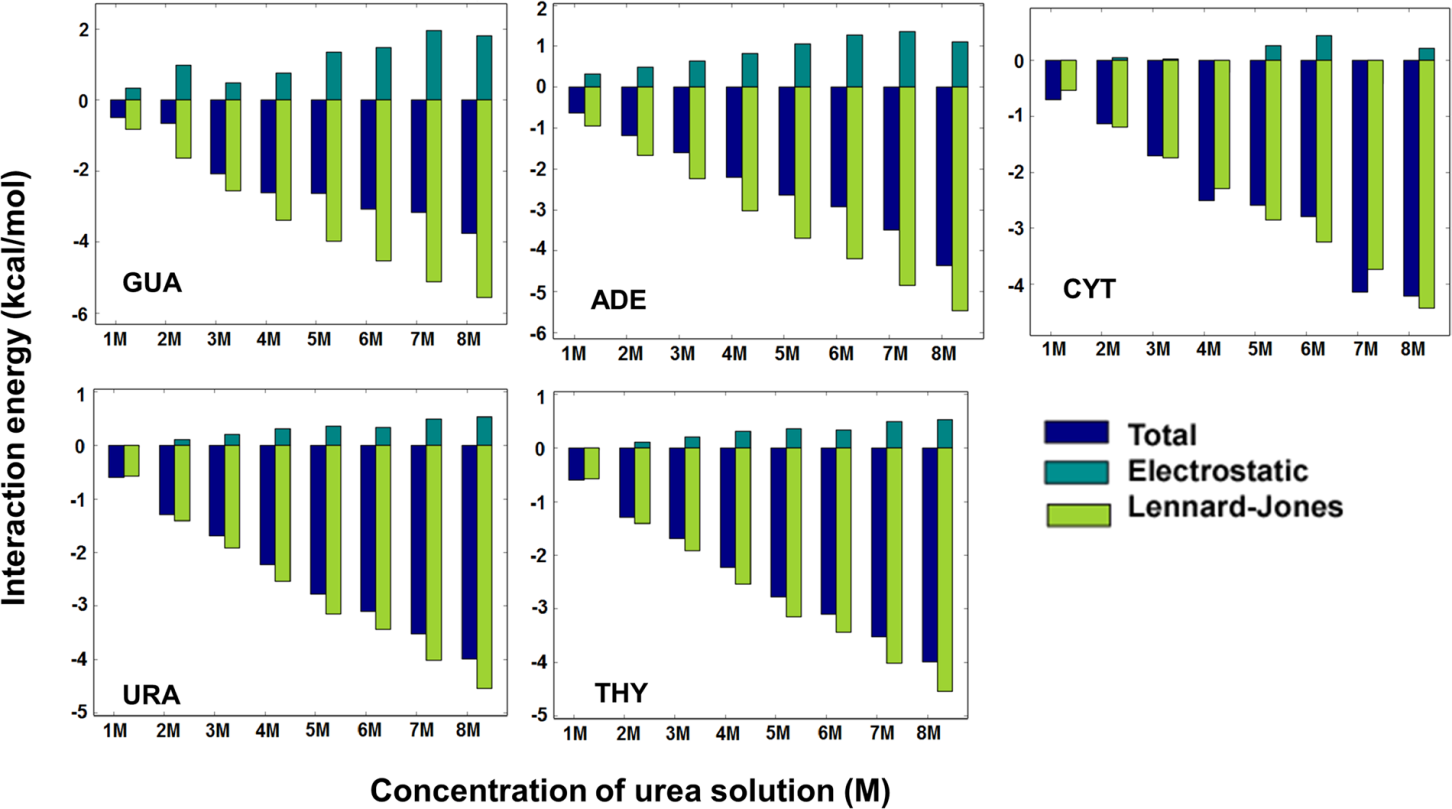
Relative mean interaction energies between nucleobases and entire solvent, and the corresponding electrostatic and LJ component energies with respect to aqueous solution (0M).

Unfolding of the nucleic acids in aqueous urea leads to exposure of the nucleobases to the solution^28^. Once the nucleobases are in their extrahelical state, they are exposed to the environment and are stabilized in the presence of urea than in its absence. The energetic basis of the preference of urea to the solvent-exposed state of the nucleobases compared to intrahelical states is analyzed here. Trajectories obtained from last 40 ns from the 45 simulations were considered for further analysis. Convergence of the interaction energies was found after the first 10 ns of the simulations due to the fact that the solvent boxes were pre-equilibrated and the nucleobases do not have any conformational degrees of freedom. The interaction energies between the base and the entire solvent environment and the corresponding contributions from the electrostatic and LJ terms of the force field were calculated. The overall interaction energies are dominated by the electrostatic interactions compared to the dispersion type interactions^35^. This higher magnitude of the electrostatic component is due to interactions between urea oxygen donor and base hydrogen acceptor atoms or base oxygen donor and urea hydrogen acceptor atoms. However, a comparison of the trends of how these interaction energies change with respect to the concentration of urea is more appropriate. Hence, relative interaction energies with reference to pure water system were calculated to understand the behavior of model systems in increasing concentration of urea (Fig. 1). The total interaction energy becomes more favorable with respect to increase in the concentration of urea in all five systems indicating that the exposure of nucleobases to the solvent environment is more favored in the presence of urea. The electrostatic contribution to the total energy is slightly repulsive or negligibly favorable with respect to the increase in the urea concentration. However, the LJ component contributes to almost all of the change in the energy due to increasing concentration of aqueous urea. This suggests that the stabilizing nature of nucleobases in their extrahelical conformation in aqueous urea is largely due to dispersive type interactions between urea and nucleobase. Notably, the primary component of the difference of the interaction energies of unfolded and folded states of protein with urea was reported to be dispersion type interactions^18,37–40^. Significantly lower dispersive type interactions in pure water explain the denaturation of RNA in the presence urea. Interaction energy analysis revealed favorable interactions of urea with purines (ADE and GUA) compared to pyrimidines (THY, CYT, and URA). The order of stability of urea-nucleobase interactions observed is G:Urea ≈ A:Urea > T:Urea ≈ C:Urea ≈ U:Urea. Guanine and adenine being purine bases have a larger hydrophobic π-face compared to the pyrimidine bases. Since the interaction energy suggests that the interaction between the nucleobases is largely due to LJ interactions where the electrostatic component is repulsive, the above order is understandable. Among the pyrimidine bases, thymine exhibits marginally more favorable interactions than the other two pyrimidines possibly due to the extra methyl group compared to the other two. These results are consistent with the other experimental studies and also our results strongly suggest that urea interactions with bases are primarily through dispersive modes^27,29^. The interaction energies presented in this section do not include the entropic contributions and hence thermodynamic integration free energy calculations were performed details of which are presented in the following section.

### Solvation free energy analysis

**Figure 2:**
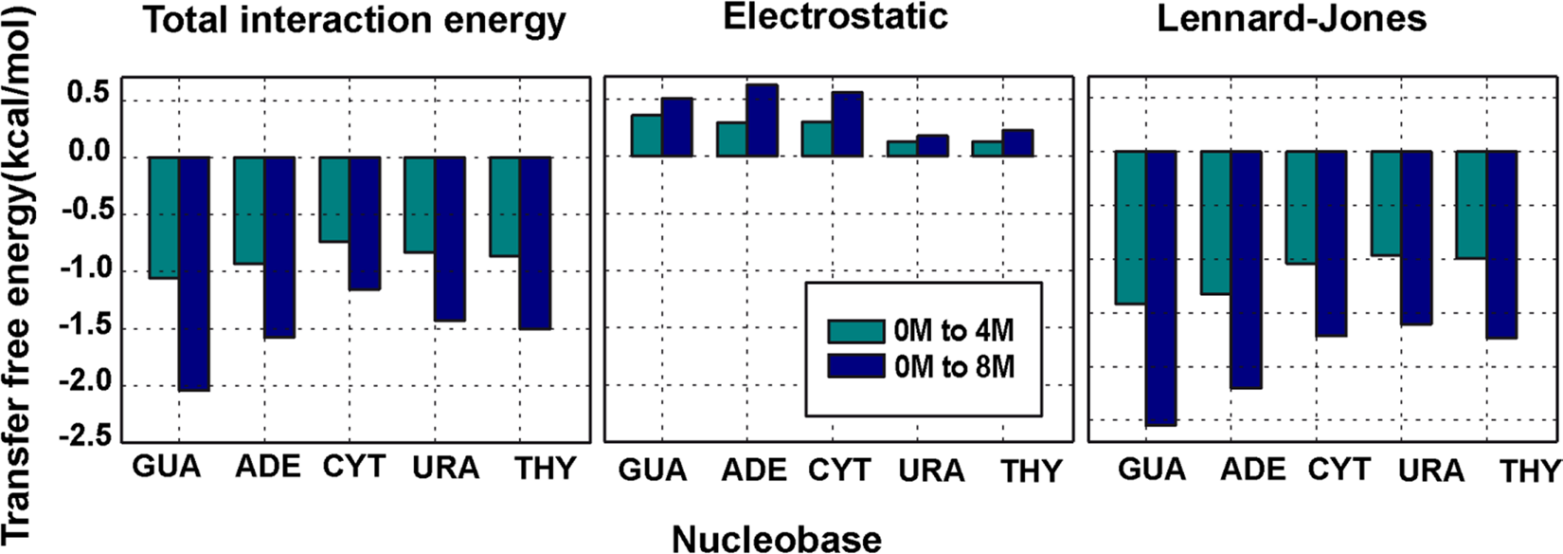
Transfer free energies from pure water to 4M and 8M urea.

Transfer free energies for the nucleobases going from water to 4M aqueous urea and water to 8M aqueous urea were calculated using thermodynamic integration method and are given in Fig. 2. Negative values of free energies indicate favorable interactions of urea with all the nucleobases. Solvation free energy analysis with changing environment showed more favorable dispersion interactions than coulombic interactions. These results are in agreement with interaction energy analysis discussed above, and also the order of stability of nucleobases is preserved when dominant Lennard-Jones component is considered. Free energy of the system takes into accounts both enthalpy and entropy component of the system^41^. Enthalpy of the system is given by ΔH = ΔU + P∆V, where P∆V term is negligible (constant pressure ≈ 1 atm and very small change in volume), therefore we can approximate enthalpy ≈ interaction energy. The contribution to the transfer free energy due to enthalpy would be same for 0M to 4M urea and for 4M to 8 M urea as there is a linear relationship between total interaction energy (≈enthalpy) and urea concentration. However, we observe that the magnitude of transfer free energy from 4M to 8M urea is less when compared to that of 0M to 4M urea (Supplementary Fig. S1). This decrease is most likely due to the differential entropic component of free energy. Initially, as urea molecules replace water molecules in the solvation shell of the solute molecules there is an entropic gain. This gain is due to the replaced water molecules being free in the bulk of the solution. However, as the concentration of urea increases and the solvent become more viscous. We have calculated the diffusion coefficient of the nucleobases in each concentration of urea solution, which is inversely proportional to the viscosity of the solution. The figure for the diffusion coefficient of nucleobase in different concentration of urea solution is provided in Fig. S2 of the Supplementary Information. The self-diffusion coefficient of the nucleobase decreases with the increase in concentration of urea solution; hence solution becomes more viscous which can be correlated from the Stokes-Einstein equation^42,43^. This supports our hypothesis that viscosity of the solvent increases with the increase in concentration of urea. This entropic gain of releasing water molecules from the solvation shell of the base molecule to the bulk is negated. The Adam’s Gibbs relation connects the viscosity (η) of a glass-forming liquid to its configurational entropy (S_c_) given by ${\rm{\eta }}={\rm{\eta }}0+[\frac{A}{TSc}]$^44^. This equation supports our observation that with increase in urea concentration there is entropic loss which accounts for the non-linear dependence of transfer free energy on the concentration of urea. The convergence of forward and backward reactions can be confirmed from the magnitude of values^45^ shown in Supplementary Table S1. Both the interaction energy and free energy calculations clearly suggest that dispersive type interactions due to the presence of urea are primarily responsible for stabilization of the nucleobases in their solvent-exposed states. Notably the guanine transfer free energy is more favorable than that of adenine though their interaction energies were similar, which possibly leads to greater preferential interaction coefficients for guanine (see below). The structural analysis of the preferential interaction of urea vs. water with the nucleobases is presented in the next section.

### Preferential interactions of urea with nucleobases compared to pure water

Interaction energy and transfer free energy analysis revealed favorable interactions of aqueous urea with nucleobases relative to pure water. Preferential interaction coefficients were calculated to examine the structural factors behind this effect^46^. Preferential interaction coefficient is experimentally measured using various techniques such as vapor pressure osmometry and dialysis which is a measure of change in chemical potential of biomolecules in response to the cosolvent^47^. Preferential interactions of urea with nucleobases relative to their interactions with water were calculated computationally using a two-domain model, which is used for extremely dilute solutions described in Eq. 1 which explains how favorably biomolecules interact with the cosolvent^46,48^.

1 $${\rm{\Gamma }}=\langle {N}_{urea}^{local}-(\frac{{N}_{urea}^{bulk}}{{N}_{water}^{bulk}}){N}_{water}^{local}\rangle $$

**Figure 3:**
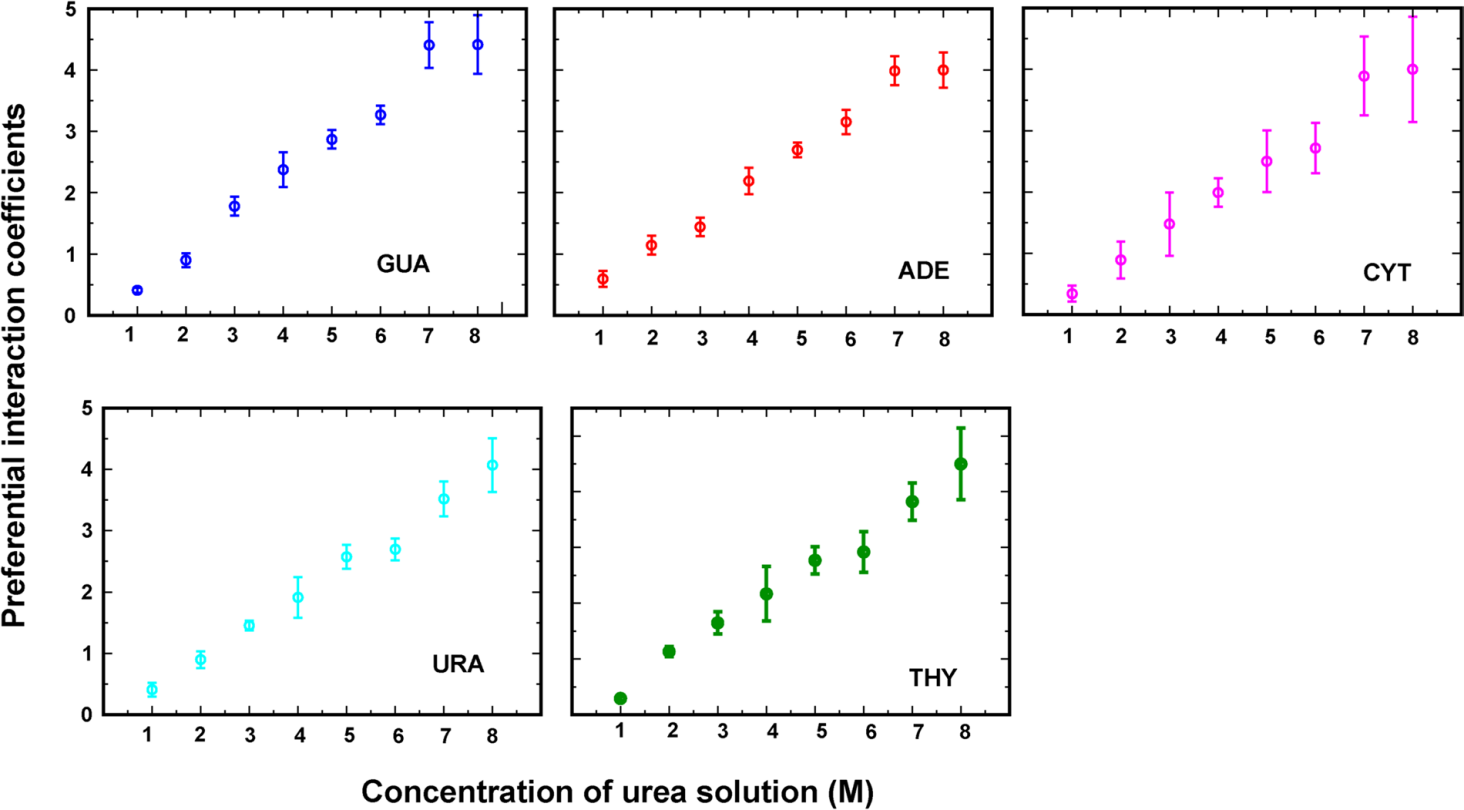
Preferential interaction coefficient calculated for all model systems using two-domain model.

Here, ${N}_{water}^{local}/{N}_{{\rm{urea}}}^{local}$ is the number of urea or water molecules in the local region i.e. within the distance of 4.5 Å from nucleobases and ${N}_{water}^{{\rm{bulk}}}/{N}_{{\rm{urea}}}^{bulk}$ is the number of urea or water molecules outside of above mentioned local region i.e. in the bulk region. The preferential interaction coefficients for all the nucleobases show the linear dependence on concentration(Fig. 3). The increase in positive values of Γ with respect to increasing concentration of urea provide valuable information about how favorably urea can interact with nucleobases over water. With increasing concentration more urea molecules interact with base moiety restricting water molecules to come in contact.

In the two-domain model, the effect of long-range electrostatic and dispersive forces are ignored while Kirkwood-Buff (K-B) integrals take into account the entire solvent and are used to derive thermodynamic properties with volume integration of the radial distribution functions (RDFs) over box volume^49–51^. K-B integrals can be obtained from NVT/NPT simulations and with the help of these integrals preferential contact coefficient can be calculated. Due to finite-size scaling effects, the RDFs for closed systems do not converge to 1 at bulk^52–54^. The RDF basically is a measure of 2-particle correlations in the system. The accuracy of the force field in modelling interactions decreases at larger distances. In order to compensate for this, we employ long-range modelling^55^. At large distances, the behavior of different interaction potentials don’t change much and hence can be assumed to be same as that of a simple Lenard-Jones fluid. Local fluctuations were removed by applying smoothing filters (Savitzky Golay filter). At long ranges, systems are assumed to behave as a Lennard-Jones fluid as interaction potential (r_i_ − r_j_), between a particle i of solute and a particle j of solvent becomes negligible. The indirect part of the RDF was calculated by fitting the calculated values to the corresponding values of a LJ fluid^55^.

2 $${{\rm{g}}}_{ij}^{Indirect}(r)=1+a.{e}^{-b(r-c)}\,\ast \,sin(d(r-e))$$

The calculated indirect RDF was fit to the above equation in the least squares sense with starting parameters as

$$\begin{array}{rcl}{a}_{init} & = & {{\rm{g}}}_{ij}({r}_{max2})\,\mbox{--}\,1,\\ {b}_{init} & = & \frac{-1}{{r}_{min2}-{r}_{max2}}ln(\frac{1-{{\rm{g}}}_{ij}({r}_{min2})}{{{\rm{g}}}_{ij}({r}_{max2})-1}),\\ {c}_{init} & = & {r}_{max2},\,{d}_{init}=\frac{\pi }{{r}_{u4}-{r}_{u3}},\,{e}_{init}={r}_{u3},\end{array}$$

here, *r*_*max*2_ is the second maxima, r_min2_ is the second minima, r_u3_ is the third time g_ij_ = 1, r_u4_ is the fourth time g_ij_ = 1. A cutoff of *r*_*u*3_ is taken for the ${{\rm{g}}}_{ij}^{Direct}(r)$ component. Solute-water RDF was calculated between the center of mass (COM) of the solute (nucleobase) and oxygen of water. Similarly, the solute-urea RDF was calculated between the COM of the solute (nucleobase) and the carbon atom of urea. In theFig. 4a smooth lines indicate smooth curves obtained from long-range modelling obtained with Eq. 2 and dotted lines are curves calculated from MD trajectories. Ideally, the KB integrals are derived for the grand canonical ensemble (µVT)^56^. Directly integrating the radial distribution function in closed NVT/NPT systems would lead to artifacts in the calculation arising from finite-size effects due to periodic boundary conditions. Kruger *et al*. derived the finite simulation KB-integrals in hyperspheres of one, two and three dimensions and is given by^49^,

3 $${G}_{ij}={\int }_{0}^{R}({g}_{ij}({r}_{12})-1)w(x,r)dr$$

where, for three dimensional hypersphere,

4 $$w(x,r)=4\pi {r}^{2}(1-3\frac{x}{2}+\frac{{x}^{3}}{2}),\,x=\frac{r}{R}$$

This correction on the KB-integrals is applied after using long-range modeling, and contact coefficients in terms of KB-integrals were calculated as^49,57^5where, S, C and W correspond to water, solute (nucleobase), and cosolute (urea) respectively.

**Figure 4:**
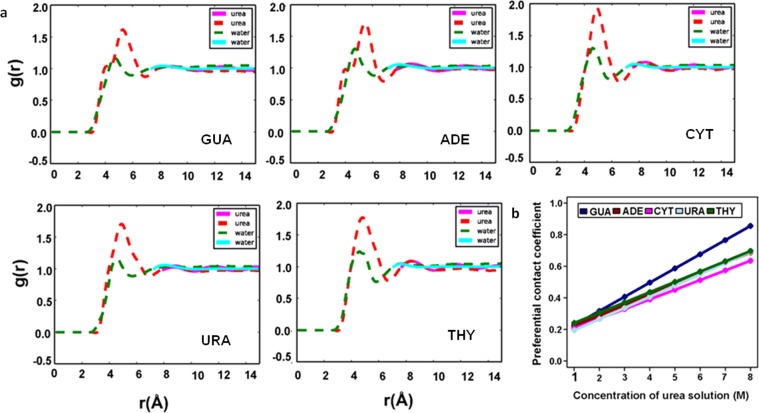
(**a**) The radial distribution function g(r) of urea and water with nucleobase in 8M concentration of urea. Smooth lines (red and green) indicate curves obtained from long-range modelling and dotted lines (pink and sky blue) are curves calculated directly from MD trajectories. (**b**) Contact coefficients calculated using K-B integrals.

RDFs obtained after applying long-range modelling (Fig. 4a and Supplementary Fig. S3) show a sharp peak of urea carbon at around 5 Å almost for all the nucleobases, while water oxygen RDFs indicates a change in urea distribution around nucleobases and water depletion around all the nucleobases. It is clear from Fig. 4a that initially, water molecules interact with nucleobases, but as the number of urea molecules increases the average probability of urea interacting with nucleobases increases. K-B integral analysis suggests a possible mechanism of intrusion of urea into nucleic acids with stronger interactions with almost all types of nucleobases compared to water indicating how urea might destabilize nucleic acid structures. Figure 4b correlates positive values of contact coefficients obtained from Eq. 5, which supports our previous observations that urea forms favorable interactions with nucleobases. The increased values of contact coefficients in higher concentrations depicts nucleobases are stable in urea compared to water. From preferential contact coefficient, we observed that nucleobases have a stronger affinity to urea molecules than water. Contact coefficients calculated considering bulk solvent shows the qualitatively similar trend as preferential interaction coefficient in which only interactions within first solvation shell was considered indicating that long-range interaction effects are negligible.

### Spatial density analysis

**Figure 5:**
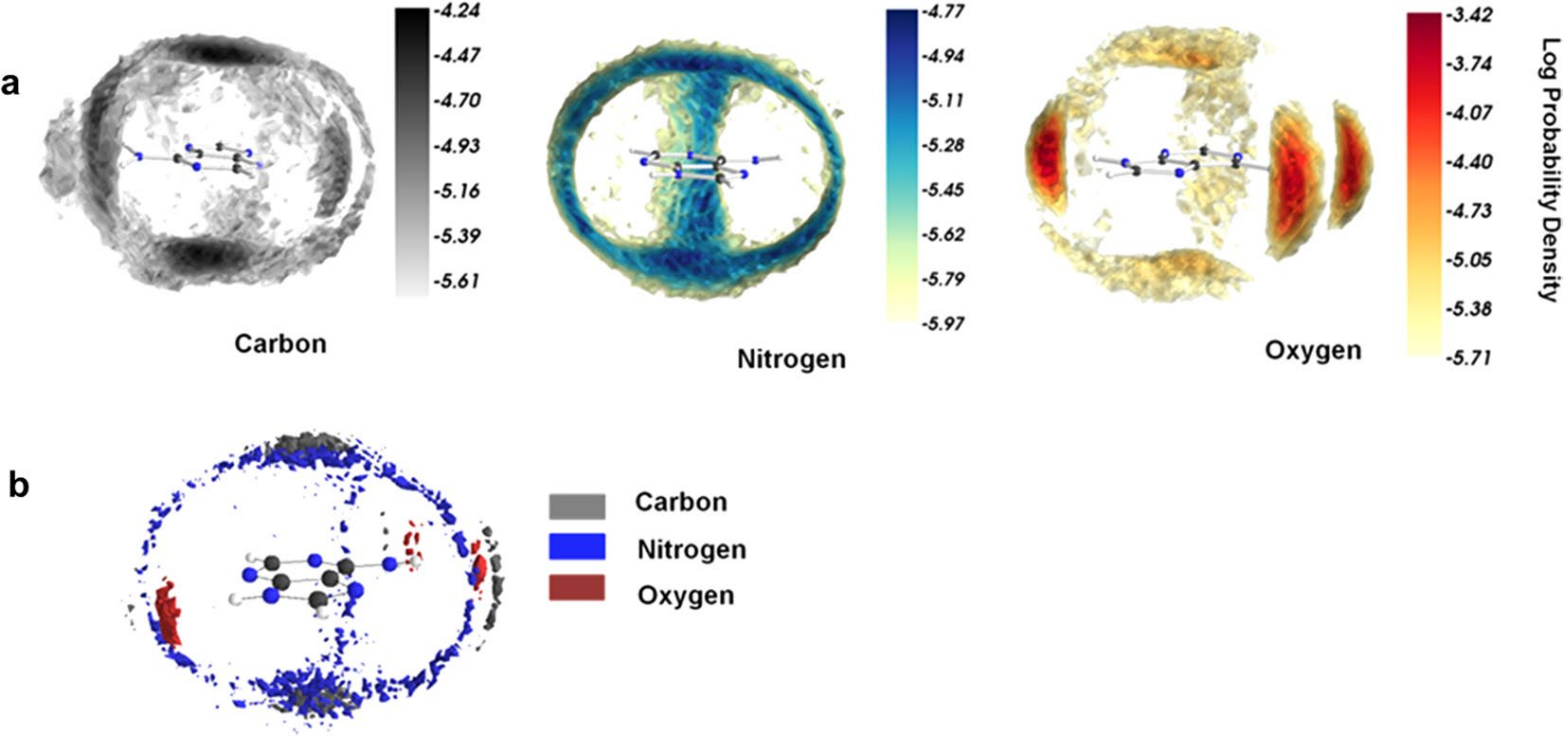
(**a**) Spatial density distribution for C, N, and O atoms of urea with respect to adenine base. (**b**) Spatial density distribution map for C, N and O atoms around adenine.

Energetic data, preferential interaction coefficients and K-B integrals analysis discussed in the previous sections confirm that urea is preferred in the vicinity of nucleobases compared to water at the molecular level. To understand the modes of interactions between urea and nucleobases spatial density distributions^58^ were analyzed based on the last 40 ns of each MD trajectory. Relative positions of the major atoms of urea(C, N, and O) with respect to the nucleobases were used to calculate spatial probabilities within a distance of 4.5 Å from any atom belonging to the model system. This forms a sphere of urea moieties of about 7.75 Å for purines and 7.05 Å for pyrimidines. This space was then divided into 75 × 75 × 75 equally spaced bins. Histogram binning was then done over this space for each C, O and N atoms to obtain their respective spatial probability densities. Spatial density maps analysis shed light on prominent interaction sites available for urea atoms around the nucleobases. In Fig. 5a, distinct high-occupancy regions for carbon, oxygen and nitrogen around the adenine molecule are observed. This reveals a structure in the way urea interacts with these base molecules. The nitrogen atom of urea forms NH-π, stacking and hydrogen bonds with nucleobase. Oxygen atoms can similarly form hydrogen bonding with donor atoms of the solute molecule. Prominent black regions of carbon atoms above and below the base molecule plane indicate that possible NH-π and π-π stacking interactions. Oxygen atoms are involved in hydrogen bonding as indicated by the deep red region near the hydrogen donor atoms of the base molecule. Figure 5b shows the isodensity surface view of carbon, oxygen and nitrogen molecules for adenine (8M urea). The corresponding data for the other four bases are given in Supplementary Fig. S4. The isosurface of carbon and nitrogen are perpendicular to the normal of the base molecule and are present above and below it. These isosurfaces are at the same level indicating that the most probable position of carbon and nitrogen lie on the same plane. This strongly suggests that urea molecules prefer possible π-π stacking interactions over NH-π or hydrogen bonding interactions. If NH-π interactions would have been preferred then we would expect the isosurface of carbon atoms to be formed above the isosurface of nitrogen depending on its position with respect to the base molecule. The following section discusses the possible modes of interactions between the nucleobases and urea that give rise to such interesting spatial density profiles.

### Three main modes of interaction between nucleobases and urea

Interaction energy, solvation free energy reveals that urea interacts with the nucleobases directly both in-plane and out-of-plane with respect to the base. Conformational stability of nucleic acids mainly results from a balance of various classes of interactions. As deciphered from spatial density maps analysis, along with conventional hydrogen bonding interactions, urea may be capable of forming NH-π and π-π interactions with nucleobases. Previous studies identified hydrogen bonding and stacking interactions as two main modes of interactions between urea and nucleobases^33,59^. We use certain geometric criteria to quantify these interactions and evaluate their respective probability distributions^37^.

### Stacking interactions

**Figure 6:**
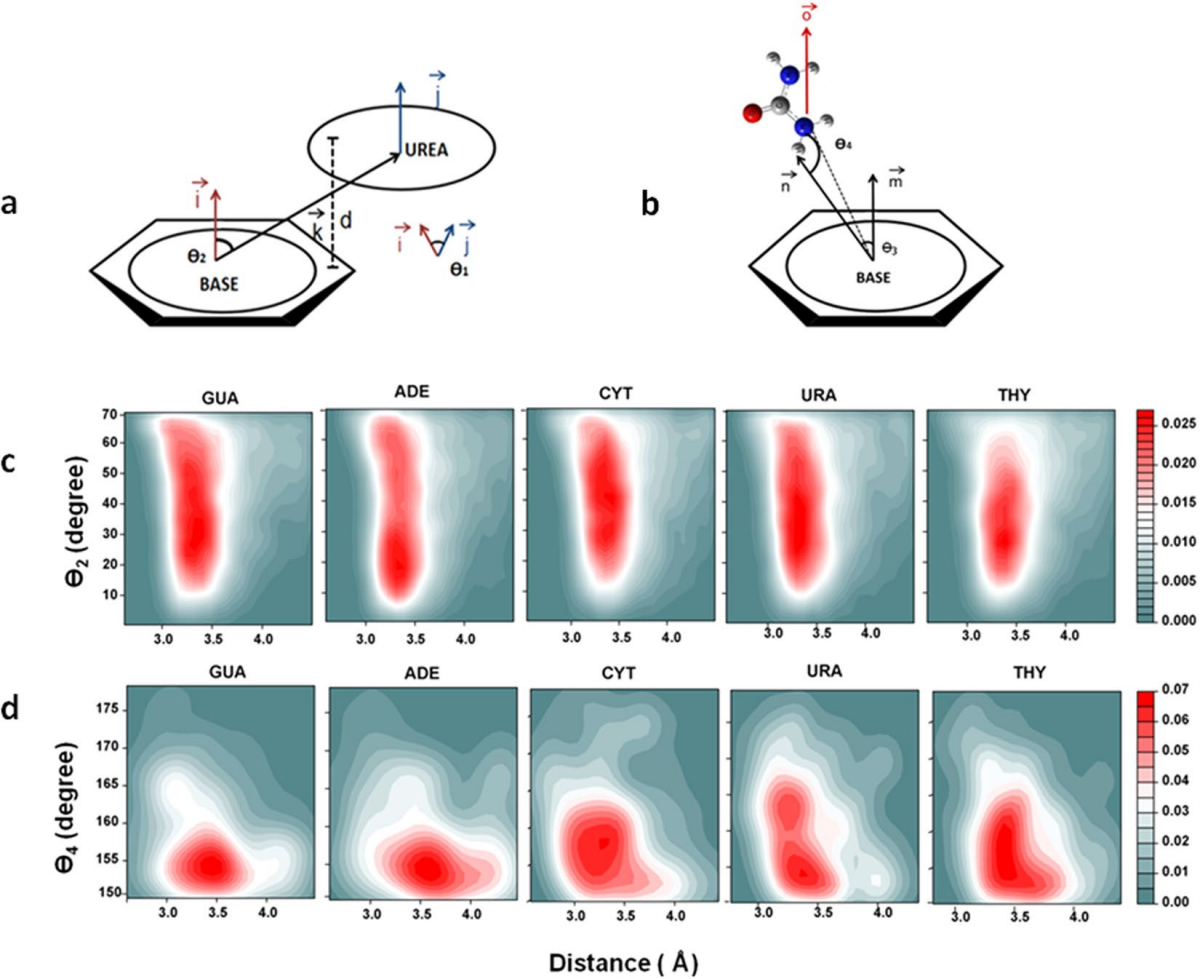
(**a**) Schematic representation of angle and distance definitions for quantifying π-π interactions. (**b**) Schematic representation of angle and distance definitions for quantifying NH-π interactions. (**c**) Probability distributions of urea molecules forming stacking interactions with nucleobase along distance d and angle θ_2_ in 8M urea. (**d**) Probability distributions of urea molecules forming NH-π interactions with nucleobase along distance d and angle θ_4_.

For π-π stacking interactions the following geometric criteria was used (1) Distance between center of mass (COM) of nucleobases and urea, d ≤ 4.5 Å; (2) Angle (θ_1_) between the vectors normal to the nucleobases and urea planes, i.e. 0 ≤ θ_1 _< 40° or 140° < θ_1_ ≤ 180° (angle between $\overrightarrow{i}$ and $\overrightarrow{j}$ in Fig. 6a) (3) Angle (θ_2_) between vector connecting COM of nucleobase and urea, and normal of nucleobase plane i.e. 0 ≤ θ_2_ < 70° or 110° ≤ θ_2_ ≤ 180° (angle between $\overrightarrow{i}$ and $\overrightarrow{k}$ in Fig. 6a). Only if all these criteria are satisfied, the urea moieties can form stacking interactions with the base molecule.

### NH-π interactions

For ΝH-π interactions, the following geometric criteria were used (1) d < 4.5 where d is the distance between COM of nucleobase and any N atom of urea. (3) Angle (θ_3_) is angle between vector normal to plane of base and vector joining COM of base to H atom of urea i.e. θ_4_ > 150° or θ_4_ < 30° (angle between $\overrightarrow{m}$ and $\overrightarrow{n}$ in Fig. 6b) (3) Angle (θ_4_) is angle between COM of base, H atom attached to N atom and N atom i.e. θ_3_ > 150° (angle between $\overrightarrow{n}$ and $\overrightarrow{o}$ in Fig. 6b) Fig. 6c,d shows the probability distributions of urea molecules taking part in π−π stacking and ΝΗ-π interactions for all bases in 8M urea respectively. Red regions in the contour plots show the most probable configurations of both stacking and NH-π interactions. Probability distributions of urea molecules involved in stacking from Fig. 6c show most urea molecules are present within ~3.4 Ǻ from the nucleobase plane and the density is higher at θ_2_ ~30°–40° for almost all nucleobases. Supplementary Figs S5 and S6 presents the probability distributions at other concentrations of aqueous urea. A large number of stacking instances observed in the probability distributions indicates strong stacking interactions of urea molecules with nucleobases. For ΝΗ-π interactions urea molecules have a higher density at around ~3.8 Ǻ and θ_4_ at ~155–160° for all the nucleobases (Fig. 6d). The substantial number of urea molecules involved in stacking and ΝΗ-π interactions within given geometric criteria suggests that along with hydrogen bonding of stacking and ΝΗ-π interactions play a major role in urea-induced denaturation process of nucleic acids. In addition to the proposed stacking and hydrogen bonded interactions, we show that NH-π interactions also play a role in stabilizing the solvent-exposed nucleobases in their extrahelical states^33^.

### Dynamic properties of stacking, NH-π and hydrogen bonding interactions

The balance of different types of interactions is one of the crucial steps for stable interactions between osmolyte and solute to understand osmolyte-induced macromolecular folding. The intricate network of all interactions present in urea-nucleobases is observed in terms of stacking, NH-π and hydrogen bonding. The average lifetime values i.e. the mean duration of urea or water molecules to remain around nucleobases were calculated. Water and urea molecules compete with each other to interact with nucleobase and more than one urea molecule at a time interacts with nucleobase. The dynamics of these interactions is studied using dwell time distribution as discussed in our previous work^37^.

6 $${P}_{dwell}(t;{t}^{\ast })=\frac{1}{{N}_{dwell}}{\sum }_{i=1}^{{N}_{dwell}}\delta [{\tau }_{i}({t}^{\ast })-t]$$

N_dwell_ is the number of events, t* is transient disruption time which is disruption period where urea molecule goes away and comes in contact again, τ is time interval of i^th^ interval and *δ*[τi(t*) − t] = 1 if t < τ_i_ t^*^) < (t + dt) where dt is time period else it is 0. Survival probability for given t* values survival probability of interaction calculated as^28,37^

7 $${\rm{S}}\,({\rm{t}};{t}^{\ast })=1-{\int }_{0}^{t}P(\tau ,\,{t}^{\ast })d\tau $$

and average lifetime for one urea molecule calculated by integrating survival probability

8 $$\langle \tau ({t}^{\ast })\rangle ={\int }_{0}^{\infty }S(t;{t}^{\ast })dt$$

**Figure 7:**
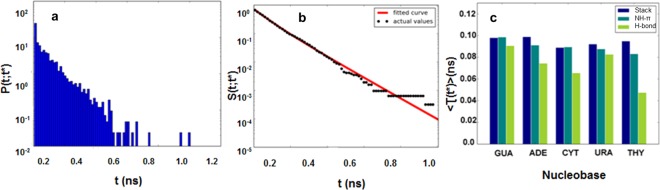
(**a**) Guanine-urea stacking dwell time distribution. (**b**) Survival probability and triexponential fit. (**c**) Comparison of average life time of guanine-urea Stacking, NH-π and Hydrogen bond interactions.

Results for 8M concentration are shown in Fig. 7a,b for guanine for most prominent stacking interactions. Dwell time distribution obtained with t* value of 0.5 ns (Fig. 7b) shows the exponential decay in survival probability with respect to increase in duration of the event. The dwell time distributions and survival probability data for the other four bases are given in the Supplemental Fig. S7. Urea maintains contact with nucleobase longest for 1 ns as longest dwell event found for ~1 ns (Fig. 7a). Mean lifetime values of all five nucleobases at t* value of 0.1 ns are compared for all three interactions i.e. stacking, NH-π and hydrogen bonding. Figure 7c depicts the mean lifetime of urea stacking interactions > lifetime of urea NH-π interactions > lifetime of urea hydrogen bonding interactions considering at least one urea molecule taking part in the respective interactions. Favorable dispersion interactions result into large values of mean time distribution for purines compared to pyrimidines. This confirms that stacking interactions formed by urea with the base are reasonably long lasting compared to NH-π and hydrogen bonding and hence plays a major role in stabilizing the solvent-exposed nucleobases.

## Conclusions

This study reports a systematic study on the possible modes of interactions between nucleobases and urea, and their structural, dynamic and energetic aspects that make the solvent-exposed states of nucleobases more stable in the presence of urea than in its absence. All atom molecular dynamics simulations and free energy calculations were performed on the five nucleobases each in nine different environments to address the above. Interaction energies and transfer free energies reveal favorable interactions with increasing concentration of urea. Examination of the electrostatic and LJ components of the energies indicates that the stabilization is primarily due to dispersion interactions as has been observed in case of proteins as well^10,37,39^. Urea forms stronger interactions with purines compared to pyrimidines due to the larger π-surface in the former. Preferential interaction coefficients calculated from the two-state model and K-B integrals indicate a strong preference for urea to be in the vicinity of the bases compared to water, and it increases linearly with respect to the concentration of aqueous urea. Spatial density profiles reveal probable in-plane and out-of-plane modes of interaction between nucleobase and urea. Detailed analysis this indicates that in addition to hydrogen bonding and π-π stacking and NH-π interactions play a major role in favorable urea-nucleobase interactions. Among the three modes of interactions, urea-nucleobase stacking interactions were found to be long lasting. Therefore, this study provides a fundamental understanding of the nature and modes of nucleobase-urea interactions that stabilize the solvent-exposed extrahelical states of nucleobases of unfolded RNA in aqueous urea. With these conclusions, we propose molecular level atomic insights on the mechanism of urea-induced denaturation process. Urea and water compete with each other to form favorable interactions with nucleobases surfaces in nucleic acid structures. Urea engages nucleobases in an extrahelical state via favorable π-π, NH-π and hydrogen bonding interactions. While both urea and water are capable of forming NH-π and hydrogen bonding interactions, stacking is possible with only urea, which indicates that the ability of urea to form stacking interaction dominated by dispersion makes it a most competent denaturant. Denaturant effects of urea may vary depending on the base composition and chemical nature of nucleic acids. Further systematic studies on the unfolding of RNA in the presence of aqueous urea are expected to provide more insights on the unfolding phenomenon.

## Computational Methods

### MD simulations of nucleobases in different environments

**Figure 8:**
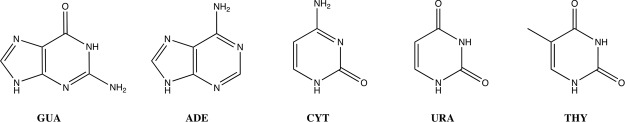
DNA and RNA bases considered in this study.

A total of 45 systems were initially generated (five nucleobases, GUA, ADE, CYT, URA and THY (Fig. 8), in water (0M) and different concentrations of urea: 1, 2, 3, 4, 5, 6, 7 and 8M) on which MD simulations have been performed. All the simulations were performed using the NAMD^60,61^ program with all-atom CHARMM36^62–64^ force field using the TIP3P^65^ water model. Pre-equilibrated solvent boxes of dimensions 32 Å × 32 Å × 32 Å were generated for each of these concentrations. Further, optimized structures of model systems (nucleobases) obtained after minimization was inserted into these pre-equilibrated solvent boxes and those water/urea molecules whose non-hydrogen atoms within 2.4 Å of any non-hydrogen atoms of the base were deleted. All the equilibration runs for urea-nucleobase systems were performed with periodic boundary conditions using the time step of 2 fs in NVT ensemble for 1 ns using NAMD^61^ simulation program. This was followed by 50 ns long production simulations for all the systems in the NPT ensemble at 300 K and 1 atm pressure. The Nose-Hoover Langevin thermostat^66^ and Langevin piston^67^ were employed to maintain constant temperature and pressure of the system respectively. The covalent bonds involving hydrogen atoms were constrained using the SHAKE Algorithm^68,69^. The particle mesh Ewald summation method (PME) was used to treat long-range electrostatic interactions^70,71^. Lennard–Jones (LJ) potential was truncated at 12 Å by applying a smoothing function from 10 Å to 12 Å^72^. All the analyses were done using the CHARMM program^63^ and in-house Python scripts.

### Solvation free energy calculations

**Figure 9:**
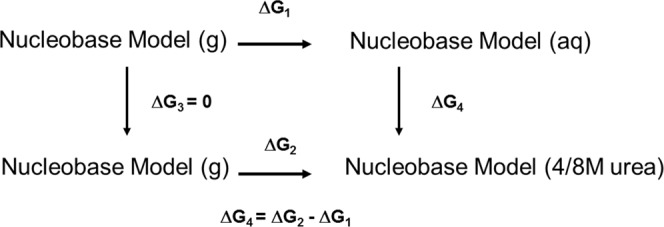
Thermodynamic cycle used to estimate transfer free energies.

Thermodynamic integration was used to quantify the change in solvation free energies of model systems with respect to change in concentration of the environment from pure water to 4M/8M urea^37^. The thermodynamic cycle used to calculate the transfer free energy is depicted in Fig. 9.

Solvation free energies were calculated using a two-step method^73^.

9 $$U(s=0,\,{\rm{g}}=0)\to U(s=1,{\rm{g}}=0)\to U(s=1,{\rm{g}}=1)$$

10 $$U(x,y,s,{\rm{g}})={U}_{x}(x)+{U}_{y}(y)+s{U}_{xy}^{LJ}(x,y)+{\rm{g}}{U}_{xy}^{Elec}(x,y)$$

Here, *U*(*x*, *y*) is the interaction energy between *x* and *y*, *U*_*x*_(*x*) is self-interaction energy of *x*, *U*_*y*_(*y*) is self interaction energy of *y*, $s{U}_{xy}^{LJ}$ and ${\rm{g}}{U}_{xy}^{Elec}$ are LJ and electrostatics interaction energies between *x* (nucleobase) and *y* (urea) at aqueous solution phase and gaseous phase respectively. Electrostatic interactions were switched on after the Lennard Jones potential’s scaling factor (s) became equal to unity. Soft-core potentials were used in place of the standard LJ potentials to prevent “end-point catastrophes”^74,75^. Both the parameters were coupled to λ which was varied from 0 to 1. For every value of λ, an equilibration run was performed for 100 ps followed by 600 ps production run. Solvation free energies were calculated using Eq. 11, in which the value of λ is increased gradually from 0.01 to 1.0 by interval of 0.01 for adequate sampling. According to the thermodynamic integration method, the free energy change for a transition from state A (gaseous state of nucleobase) to state B (nucleobase completely solvated in a 0M/4M/8M urea solvent) is given by the following equation^75,76^

11 $${{\rm{\Delta }}G}_{solv(A\to B)}\to {\int }_{0}^{1}{\langle \frac{\partial U(\lambda )}{\partial \lambda }\rangle }_{\lambda }d\lambda $$

In Eq. 11, λ = 1 denotes that the model system is immersed in the solution (aqueous phase) and when λ = 0 the model system is in the gas phase. Simulations for both backward and forward reactions with a cumulative time of 1.8 µs were performed to ensure convergence of the method. In case of backward reactions, a value of λ = 1 represents the model system in the gaseous state and a value of λ = 0 represents the solvated state of the model system. Other details of the analysis are given in the results and discussion section.

## Supplementary information

Supplementary Information
