# Supplementary material for: Energetic, Structural and Dynamic Properties of Nucleobase-Urea Interactions that Aid in Urea Assisted RNA Unfolding
Source: Sci Rep. 2019 Jun 19;9:8805. doi: 10.1038/s41598-019-45010-8 (PMC6584539; doi:10.1038/s41598-019-45010-8)
Supplement: Supplementary file 1 — Supplementary Information [file 41598_2019_45010_MOESM1_ESM.docx]

**Energetic, Structural and Dynamic Properties of Nucleobase-Urea Interactions that Aid in Urea Assisted RNA Unfolding**

Tanashree Jaganade, Aditya Chattopadhyay, Nila M. Pazhayam, U. Deva Priyakumar*

Center for Computational Natural Sciences and Bioinformatics, International Institute of Information Technology, Hyderabad, 500032, India

* Email: [deva@iiit.ac.in](mailto:deva@iiit.ac.in)

Phone: +91 40 6653 1161

FAX: +91 40 6653 1413


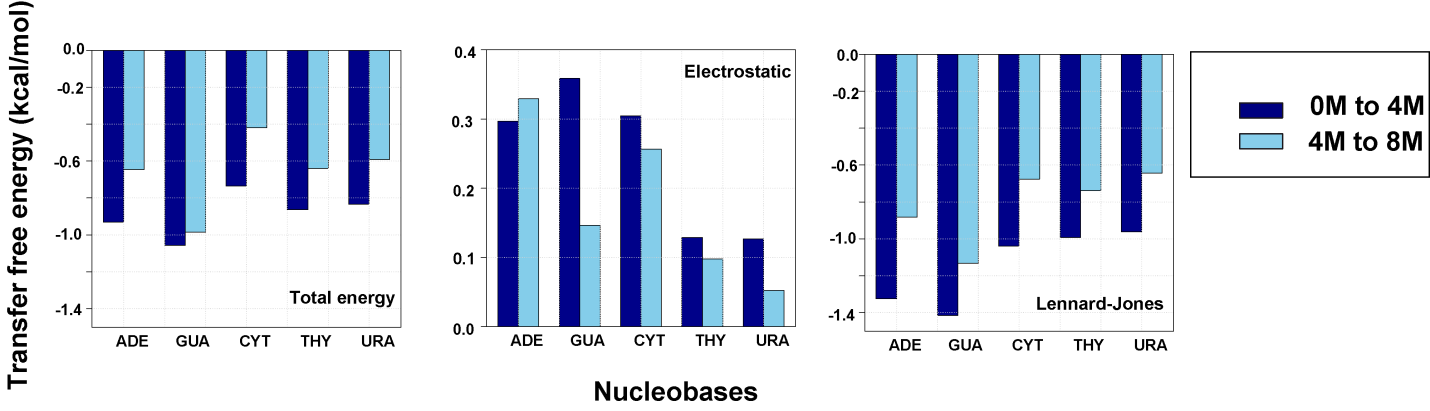


**Supplementary Figure S1:** Transfer free energies from pure water to 4 and 4M to 8 M urea

**Supplementary Figure S2:** Self-diffusion coefficient of solute (nucleobases) with respect to urea concentration.

Self-diffusion coefficient was calculated using following equation:

$\left| \left\langle r_{i}\left( t \right)-r_{i}\left( 0 \right) \right\rangle^{2} \right|=6Dt$ (1)

Here, $\left| \left\langle r_{i}\left( t \right)-r_{i}\left( 0 \right) \right\rangle^{2} \right|$ is mean square displacement of solute (Nucleobase), D is self-diffusion coefficient and t is simulation time. Stokes-Einstein equation gives the relation between diffusion of the particle and viscosity of the solution and is as follows:

$D=\frac{K_{B}T}{6r}$ (2)

Here, $K_{B}$is Boltzmann constant, T is absolute temperature, is viscosity and r is the radius of particle. The diffusion coefficient of the nucleobase decreases with the increase in concentration of urea solution; hence solution becomes more viscous which can be correlated from the above equation. This supports our hypothesis that viscosity of the solvent increases with the increase in concentration of urea. The Adam’s Gibbs relation connects the viscosity () of a glass-forming liquid to its configurational entropy (Sc) which supports our observation that with an increase in urea concentration there is an entropic loss which accounts for the non-linear dependence of transfer free energy on the concentration of urea.

**Supplementary Table S1:** Transfer free energies (kcal/mol) obtained for both forward and backward interactions using thermodynamic integration method.

| **Transfer free energy (forward )** | | | | **Transfer free energy** **(backward** ) | | | |
| --- | --- | --- | --- | --- | --- | --- | --- |
| **Concentration (0M)** | **TE** | **Elec** | **vdW** | **Concentration (0M)** | **TE** | **Elec** | **vdW** |
| ADE | -18.4 | -15.6 | -2.7 | ADE | 18.3 | -15.7 | -2.6 |
| GUA | -28.7 | -25.8 | -2.8 | GUA | 28.8 | -26.0 | -2.8 |
| CYT | -21.1 | -19.7 | -1.4 | CYT | 21.0 | -19.5 | -1.5 |
| THY | -12.7 | -12.0 | -0.7 | THY | 12.7 | -12.0 | -0.7 |
| URA | -14.2 | -13.1 | -1.1 | URA | 14.3 | -13.1 | -1.2 |
|  |  |  |  |  |  |  |  |
| **Concentration (4M)** | **TE** | **Elec** | **vdW** | **Concentration (4M)** | **TE** | **Elec** | **vdW** |
| ADE | -19.3 | -15.3 | -4.0 | ADE | 19.2 | -15.3 | -4.0 |
| GUA | -29.7 | -25.5 | -4.2 | GUA | 29.7 | -25.6 | -4.2 |
| CYT | -21.9 | -19.4 | -2.4 | CYT | 21.8 | -19.3 | -2.5 |
| THY | -13.6 | -11.9 | -1.7 | THY | 13.6 | -11.9 | -1.7 |
| URA | -15.1 | -13.0 | -2.1 | URA | 15.2 | -13.0 | -2.1 |
|  |  |  |  |  |  |  |  |
| **Concentration (8M)** | **TE** | **Elec** | **vdW** | **Concentration (8M)** | **TE** | **Elec** | **vdW** |
| ADE | -20.0 | -15.0 | -4.9 | ADE | 19.9 | -15.1 | -4.8 |
| GUA | -30.7 | -25.3 | -5.4 | GUA | 30.5 | -25.2 | -5.4 |
| CYT | -22.3 | -19.2 | -3.1 | CYT | 22.4 | -19.2 | -3.2 |
| THY | -14.2 | -11.8 | -2.5 | THY | 14.3 | -11.8 | -2.5 |
| URA | -15.7 | -13.0 | -2.7 | URA | 15.6 | -13.0 | -2.7 |


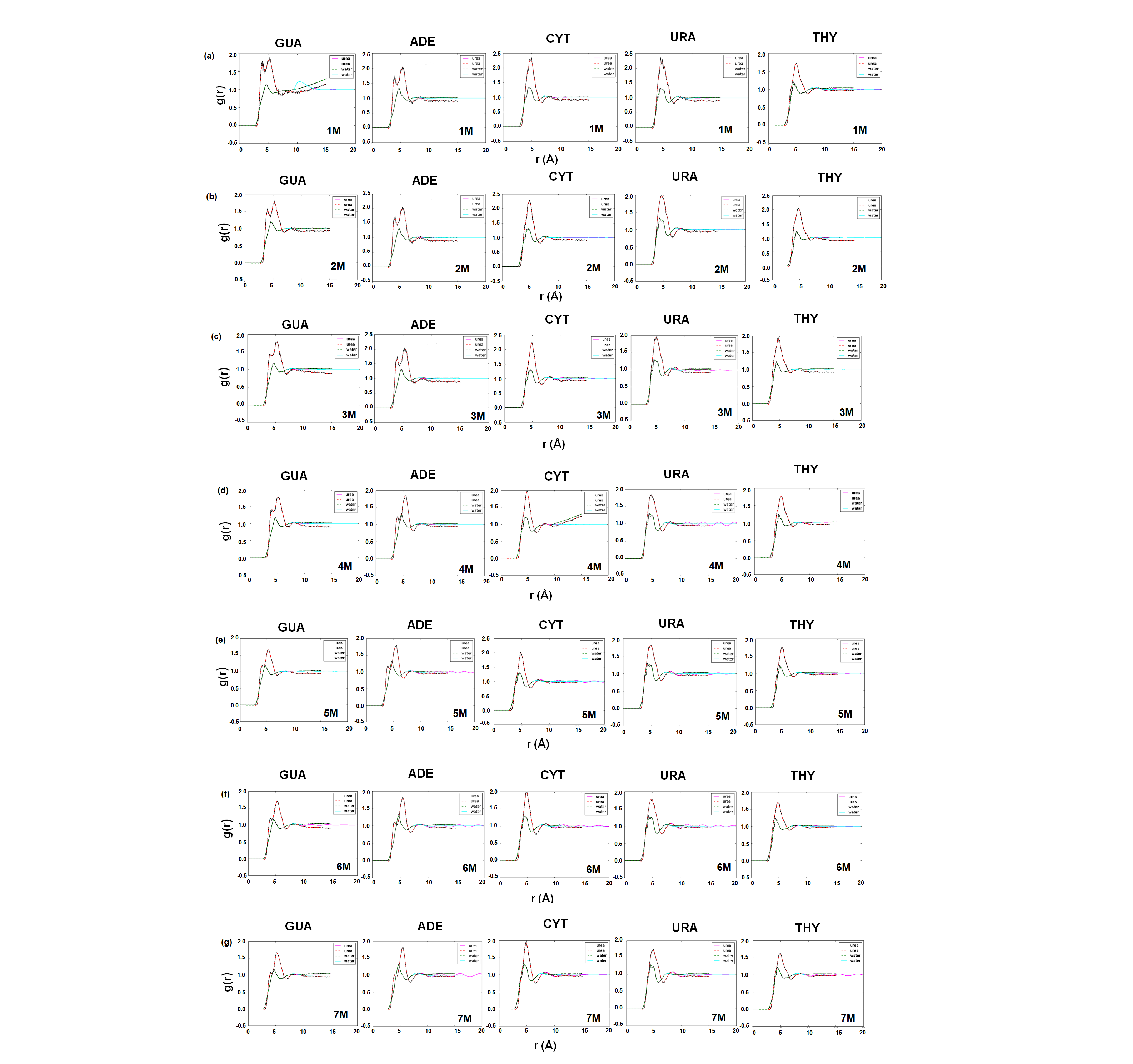


**Figure S3:** The radial distribution function **g_ij (r)_** of urea and water with nucleobase (GUA, ADE, CYT, URA and THY) in 1 to 7M concentration of urea. Dashed lines are smooth curves calculated with long-range modelling and solid lines are obtained from MD trajectories.


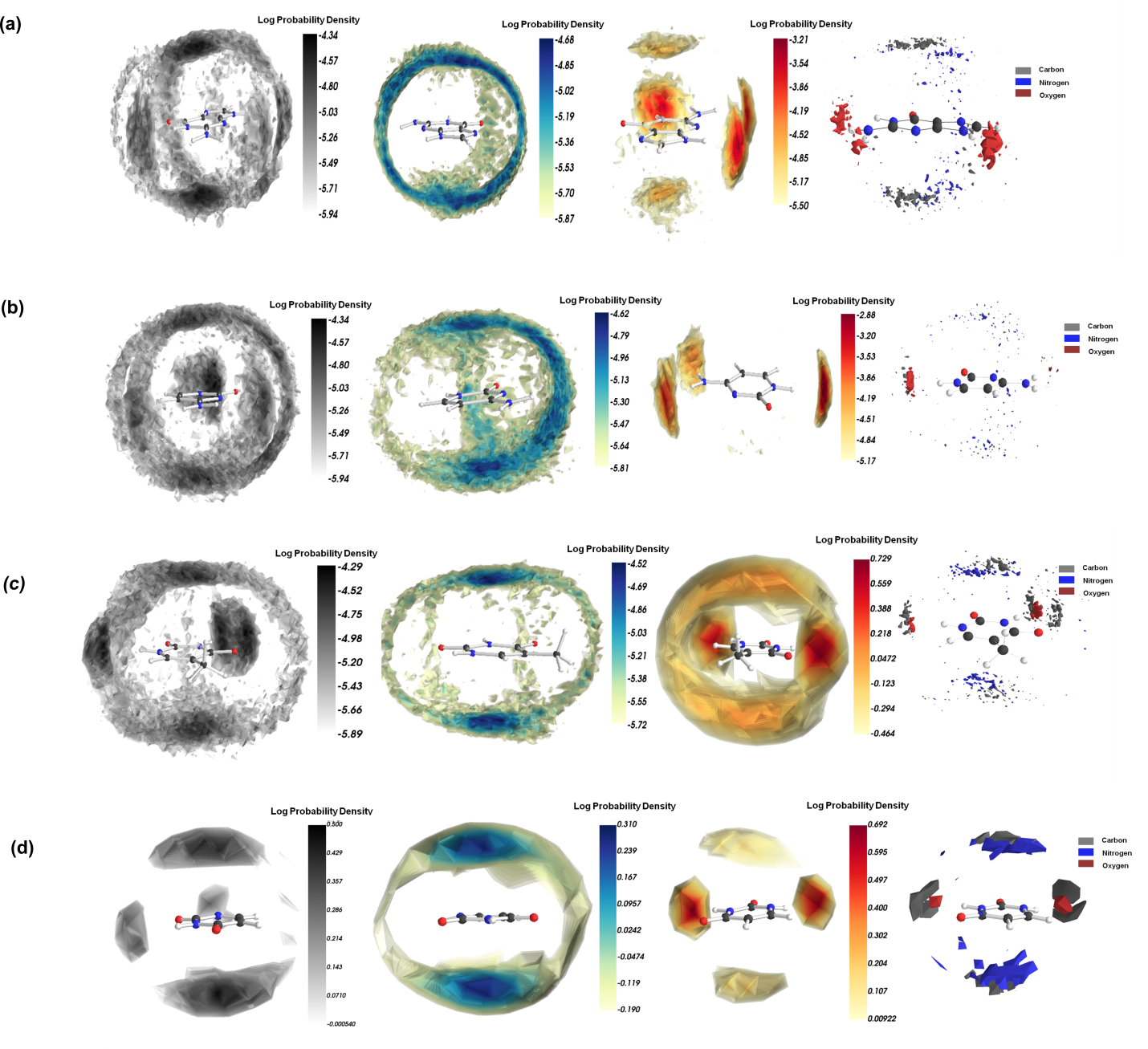


**Supplementary Figure S4:** Spatial density distribution for C, N, and O atoms of urea around GUA, CYT, URA, and THY respectively in 8M urea.


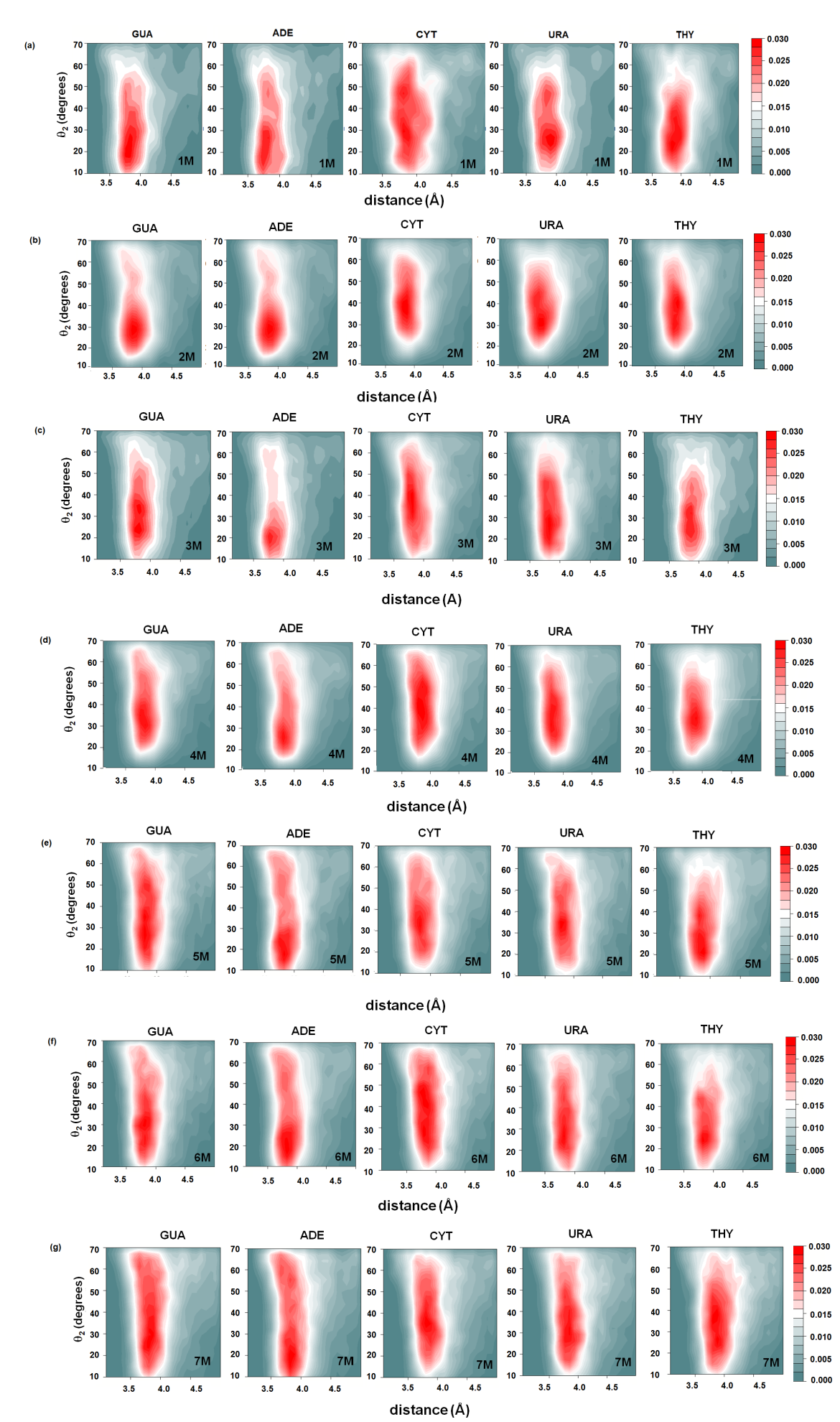


**Supplementary Figure S5:** Probability distributions of urea molecules forming stacking interactions with nucleobase along distance d and angle θ_2_ for GUA, ADE,CYT,URA and THY in 1, 2, 3, 4, 5, 6, and 7M (a to g) concentrations of urea.


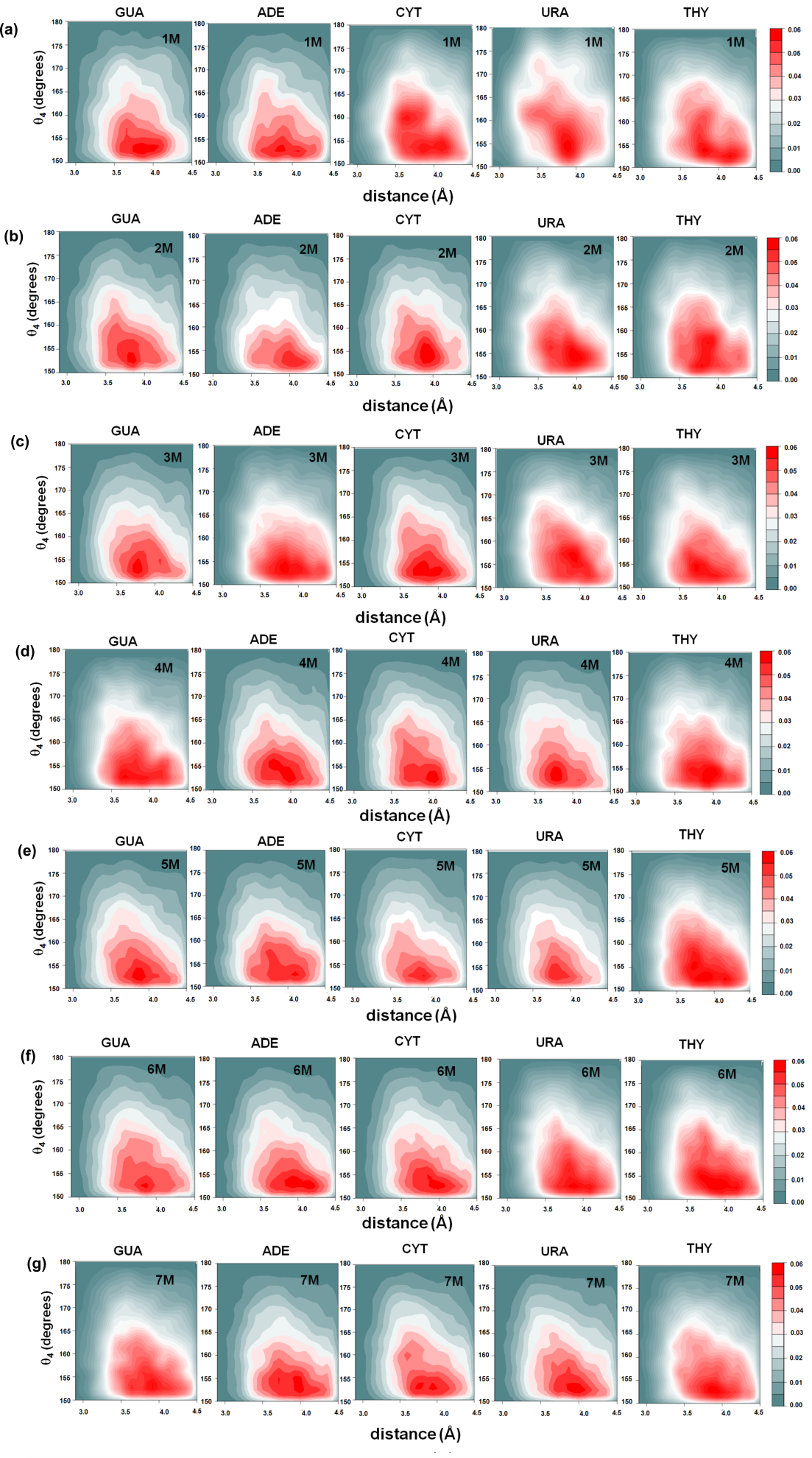


**Supplementary Figure S6:** Probability distributions of urea molecules forming NH-π interactions with nucleobase along distance d and angle θ_2_ for GUA, ADE,CYT ,URA and THY in , 2, 3, 4, 5, 6, 7, and 7M (a to g) concentrations of urea.


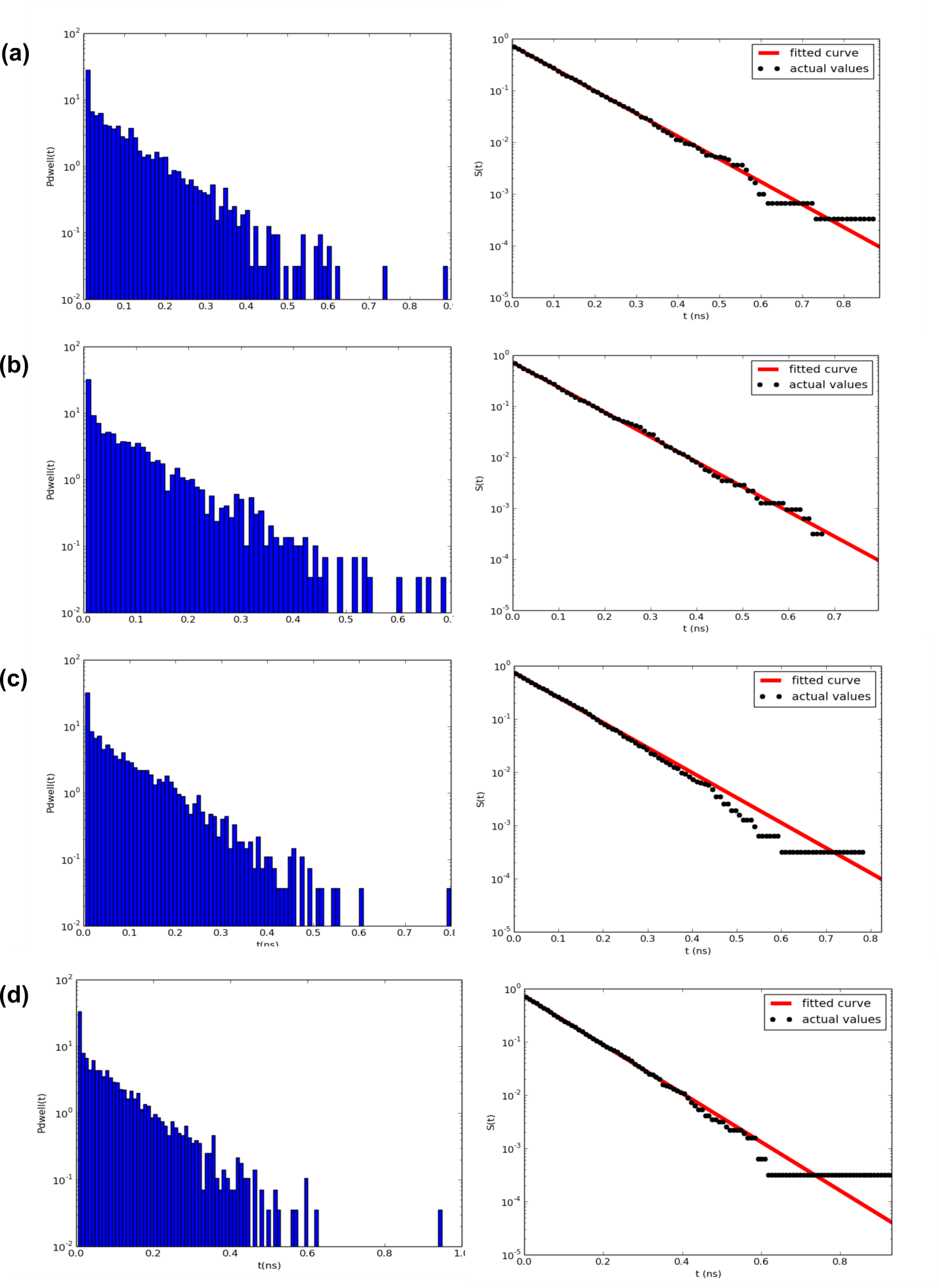


**Supplementary Figure S7:** Dwell time distribution, Survival probability and triexponential fit of ADE, CYT, URA, THY-urea stacking interactions at t*=0.5 ns.
